# Accuracy of placement of the glenoid component in reverse shoulder arthroplasty using a custom baseplate for severe glenoid deficiency

**DOI:** 10.1016/j.jseint.2024.09.013

**Published:** 2024-09-30

**Authors:** Katsumasa Nakazawa, Tomoya Manaka, Yukihide Minoda, Nobuyasu Ochiai, Yasuhiro Nakane, Yoichi Ito, Yoshihiro Hirakawa, Ryosuke Iio, Kenta Inagaki, Hiroaki Nakamura

**Affiliations:** a Department of Orthopaedic Surgery, Osaka Metropolitan University Graduate School of Medicine, Osaka, Japan; b Department of Orthopaedic Surgery, Chiba University Graduate School of Medicine, Chiba, Japan; c Sumiya Orthopedic Hospital, Wakayama, Japan; d Osaka Shoulder Center, Ito Clinic, Osaka, Japan; e Ishikiriseiki hospital, Higashiosaka, Japan

**Keywords:** Shoulder arthroplasty, Severe glenoid bone defect, Implant failure, Component placement, Glenoid version, Glenoid inclination, Custom-made implant

## Abstract

**Background:**

Glenoid bone deficiency can lead to early component loosening and implant failure during reverse total shoulder arthroplasty (rTSA). Recently, the glenoid Vault Reconstruction System (Zimmer-Biomet, Warsaw, IN, USA), a computer-aided design ot computer-assisted manufacturing system, was developed, with good clinical outcomes, including no radiographic loosening. This study examined the postoperative accuracy of glenoid component placement using this system at three different facilities.

**Methods:**

Nine patients undergoing rTSA with vault reconstruction system performed by three board-certified, fellowship-trained shoulder surgeons at three different institutions between August 2020 and January 2023 were included. Preoperative and postoperative computed tomography was performed, and glenoid inclination and version were measured using a postoperative three-dimensional evaluation system. Surgical time and intraoperative blood loss were also measured.

**Results:**

The range of errors of glenoid inclination and version were 3.5 ± 2.5° (0.4–8.3) and 3.2 ± 2.2° (0.4–6.7), respectively. In primary cases, the error ranges of both glenoid inclination and version were within 5° in six of seven cases (85.7%). In revision cases, both glenoid inclination and version were within 10°. The mean operative time was 131.4 ± 48.9 (80–206) min and the mean intraoperative blood loss was 161.1 ± 94.2 (30–300) ml; there were no intraoperative complications.

**Conclusion:**

In the present study, the placement position was good in primary and revision cases, making the placement of the glenoid component of the rTSA using vault reconstruction system in cases of glenoid bone deficiency highly reproducible.

Reverse total shoulder arthroplasty (rTSA) is a useful method for relieving pain and improving function in patients with irreparable rotator cuff tears, cuff tear arthropathy, proximal humerus fractures, fracture sequelae, revision shoulder arthroplasty, and severe osteoarthritis. However, several cases of glenoid bone deficiency, which are difficult to treat, have been recognized in rTSA. In particular, glenoid bone deficiency is present in cases of osteoarthritis, rheumatoid arthritis, chronic dislocation, and Milwaukee shoulder syndrome; in addition, it occurs frequently in cases of revision total shoulder arthroplasty.^2^^,^^22^

Treatment methods for glenoid bone deficiency in rTSA include eccentric reaming, bone grafting, and metallic baseplate augmentation with alternative centerline central screw placement.^10^^,^^15^ However, these surgical methods are very challenging, and their postoperative outcomes are poor owing to complications, such as implant loosening and bone resorption; therefore, treatment for glenoid bone deficiency remains uncertain.^15^^,^^16^

In recent years, using computer-aided design or computer-assisted manufacturing (CAD or CAM), patient-specific glenoid components have been utilized in cases of severe glenoid bone defects.^7^^,^^9^^,^^11^ In particular, the glenoid Vault Reconstruction System (VRS) (Zimmer-Biomet, Warsaw, IN, USA) combined with the Comprehensive Reverse Shoulder Arthroplasty System (Zimmer-Biomet, Warsaw, IN, USA) has been reported to significantly improve pain, functional outcomes, and range of motion (ROM) in primary and revision cases in the short term, with no obvious postoperative loosening, especially on plain radiographs.^7^

The position of the glenoid component is crucial for the longevity of total shoulder arthroplasty and particularly important in cases of glenoid bone deficiency. ^29^ We considered it important to evaluate whether VRS reproduced the exact implant position. However, no studies using three-dimensional (3D) imaging have evaluated postoperative VRS placement. Recently, the ZedShoulder software (Lexi, Tokyo, Japan) was introduced as a postoperative 3D evaluation system, which can automatically measure postoperative glenoid inclination (GI) and glenoid version (GV).^4^ The present study aimed to evaluate the accuracy of the placement of the glenoid component, with VRS performed at multiple centers using a 3D postoperative evaluation system. We hypothesized that the VRS could be used to reproduce good placement positions in cases of severe glenoid bone loss, even at different facilities.

## Materials and methods

### Patients

This was a retrospective case series of patients with severe glenoid deficiency treated with a custom glenoid baseplate. The implant used was a glenoid VRS combined with the Comprehensive Reverse Shoulder Arthroplasty System (Zimmer-Biomet, Warsaw, IN, USA). This study was approved by our institutional review board.

Nine cases of rTSA performed by three surgeons (TM, YN, and NO) at three different institutions between August 2020 and January 2023 were included. Seven cases involved the right shoulders and two involved the left shoulders; one patient was male and eight were females. The diagnoses were cuff tear arthropathy in five cases, chronic dislocation in two cases, and revision of cases after total shoulder arthroplasty in two cases. The mean age was 76.4 ± 8.5 (57–85) years, height, 146.9 ± 9.2 (136–159.7) cm, weight, 52.6 ± 12.4 (38–72.1) kg, and body mass index 24.2 ± 4.1 (19.2–29.5). The surgeons were board-certified, fellowship-trained shoulder surgeons with > 15 years of experience at each of the three facilities.

### Preoperative planning

A 3D model was created based on each patient's computed tomography (CT) data obtained within 6 months prior to surgery, and the engineer created a custom-made implant with reference to the base of the coracoid, acromion, and glenoid bone defects. The engineer suggested the direction and size of the implant, direction and size of the screw, and appropriate bone resection; the surgeon who performed the preoperative planning created a custom-made implant based on these suggestions.

GV and GI were determined by referring to the Friedman line connecting the medial border of the scapula and glenoid center (created from the original anterior, posterior, superior, and inferior glenoid center points.^13^

All three surgeons set the GV at 0°. Two surgeons in this study (TM and YN) set the GI at −10° and one surgeon (NO) set the target at −26° and −27°, parallel to the supraspinatus fossa that the reverse shoulder angle was 0° (Table I).^8^

**Table I:** Patient characteristics and operative time, blood loss and target, and actual placement.

Patients	Hospital	Surgeon	Age at surgery	Sex	Laterality	Diagnosis	Primary or revision	Operation time	Amount of blood loss	Target GI	Target GV	Actual GI	Actual GV
1	1	A	72	M	R	Chronic shoulder dislocation	Primary	105	180	−10	0	−15.1	5.1
2	1	A	76	F	R	Glenohumeral OA with severe glenoid dysplasia	Primary	85	180	−10	0	−8.9	−3.5
3	1	A	77	F	L	Glenohumeral OA with severe glenoid dysplasia	Primary	80	50	−10	0	−14.2	0.4
4	1	A	83	F	R	Glenohumeral OA with severe glenoid dysplasia	Primary	107	300	−10	0	−14.4	4.5
5	2	B	74	F	R	Failed TSA with glenoid component loosening	Revision	186	236	−10	0	−3.3	4.4
6	2	B	81	F	R	Glenohumeral OA with severe glenoid dysplasia	Primary	206	139	−10	0	−9.6	−0.6
7	2	B	83	F	R	Failed TSA with glenoid component loosening	Revision	192	255	−10	0	−10.6	8.3
8	3	C	57	F	L	Chronic shoulder dislocation	Primary	109	80	−26	0	−28.2	−3.5
9	3	C	85	F	R	Glenohumeral RA with severe glenoid dysplasia	Primary	113	30	−27	0	−31.1	1.3

*M*, male; *F*, female; *R*, right side; *L*, left side; *Primary*, primary case; *Revision*, revision case; *GI*, glenoid inclination (+: superior, -: inferior); *GV*, glenoid version (+: anteversion, -: retroversion); *OA*, osteoarthritis.

### Intraoperative approach

General anesthesia combined with an interscalene muscle block was administered, and the patient was positioned in the beach chair position. Intraoperative tranexamic acid was not used by two surgeons (TM and NO); however, surgeon YN used one ampule intraoperatively (10% Transamin, 10 ml, 1000 mg; Daiichi-Sankyo, Tokyo, Japan).

In all cases, a skin incision was made in a line from the coracoid process to the deltoid tuberosity of the humerus using a deltopectoral approach. Glenoid exposure was initially performed by applying a retractor to the posteroinferior glenoid margins. Afterward, a retractor was applied to the anterior glenoid, the anterosuperior capsule of the glenoid was dissected along the glenoid with an electrocautery scalpel, and the glenoid rim was removed. The inferior glenoid capsule was resected, and the inferior and posterior glenoid capsules were dissected to fully expose the glenoid.

When using an implant with a boss after full exposure of the glenoid, the boss reaming guide was properly adapted and stabilized in the glenoid. A Kirschner wire was inserted into the boss reaming guide, followed by the insertion of a 3.2-mm Steinmann pin to ensure that it was tangential to the medial cortical wall and perforated. After removal of the Kirschner wire, a cannulated boss reamer was inserted through the Steinmann pin and reamed. Afterward, the boss was complete, or in the absence of a boss, soft tissue and bone resection was performed for proper implant placement.

Considering the use of boss, if the metal part of the VRS was designed to be thinner than the standard type baseplate, boss was used; if the metal part was thicker, boss was not used.

To achieve initial implant stability, two or more 2.7 mm drills were inserted, beginning with the preassembled gold F.A.S.T. guide (Zimmer-Biomet). Thereafter, a central screw drill was used to create a 3.2-mm diameter hole. A 6.5-mm central screw of the desired length was inserted and fully tightened with a 3.5-mm hex screwdriver. Afterward, the peripheral screws were inserted using the FAST guide and a 2.7-mm drill, followed by drilling using a 3.5-mm hex screwdriver. Thereafter, a glenosphere was inserted. In all cases, the glenosphere was a 36-mm standard type.

### CT imaging analysis and virtual arthroplasty

**Figure 1:**
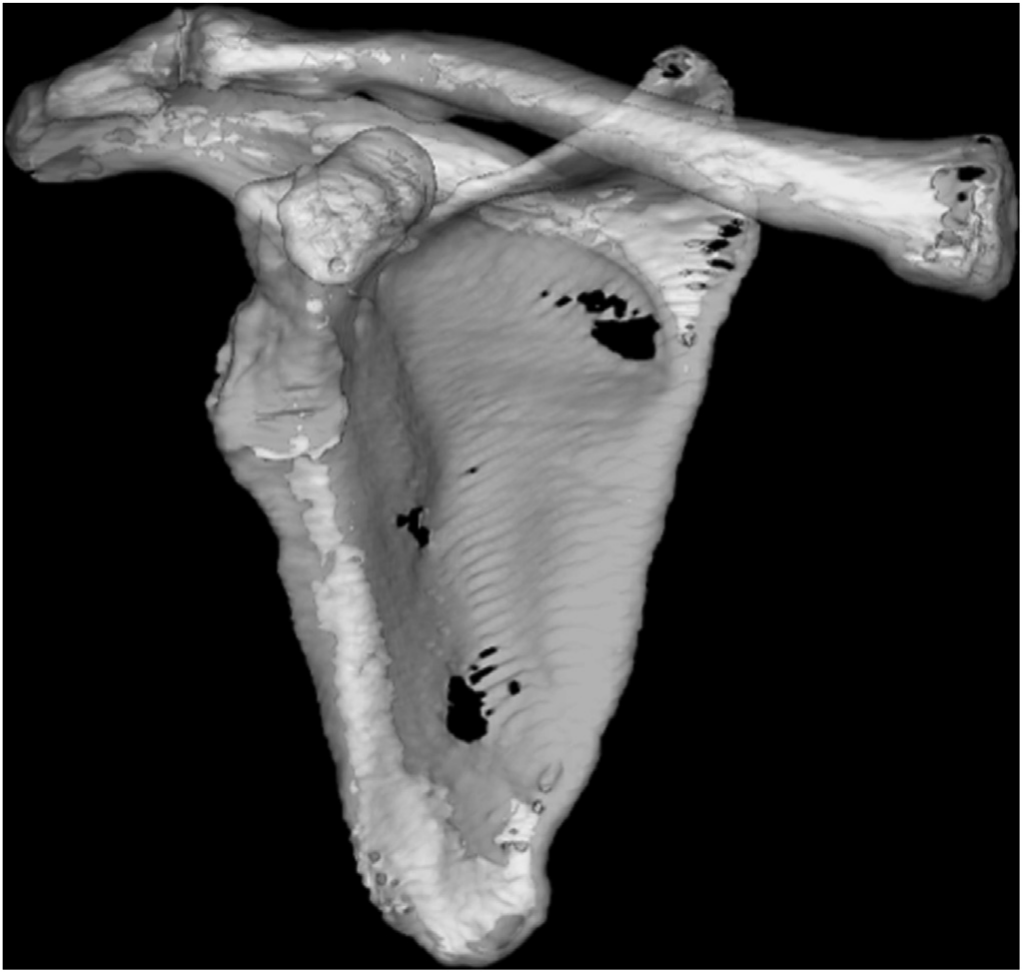
3-dimensional bone model created from preoperative computed tomography (CT) data of scapula.

CT was performed preoperatively and 1 month postoperatively. One millimeter slices of the scapula were imaged and digital imaging and communications in medicine format was used for CT imaging. Zed Shoulder software (Lexi, Tokyo, Japan) was used for the measurements; this software creates a semiautomated 3D bone model by selecting reference points based on preoperative CT data of the scapula (Fig. 1).

**Figure 2:**
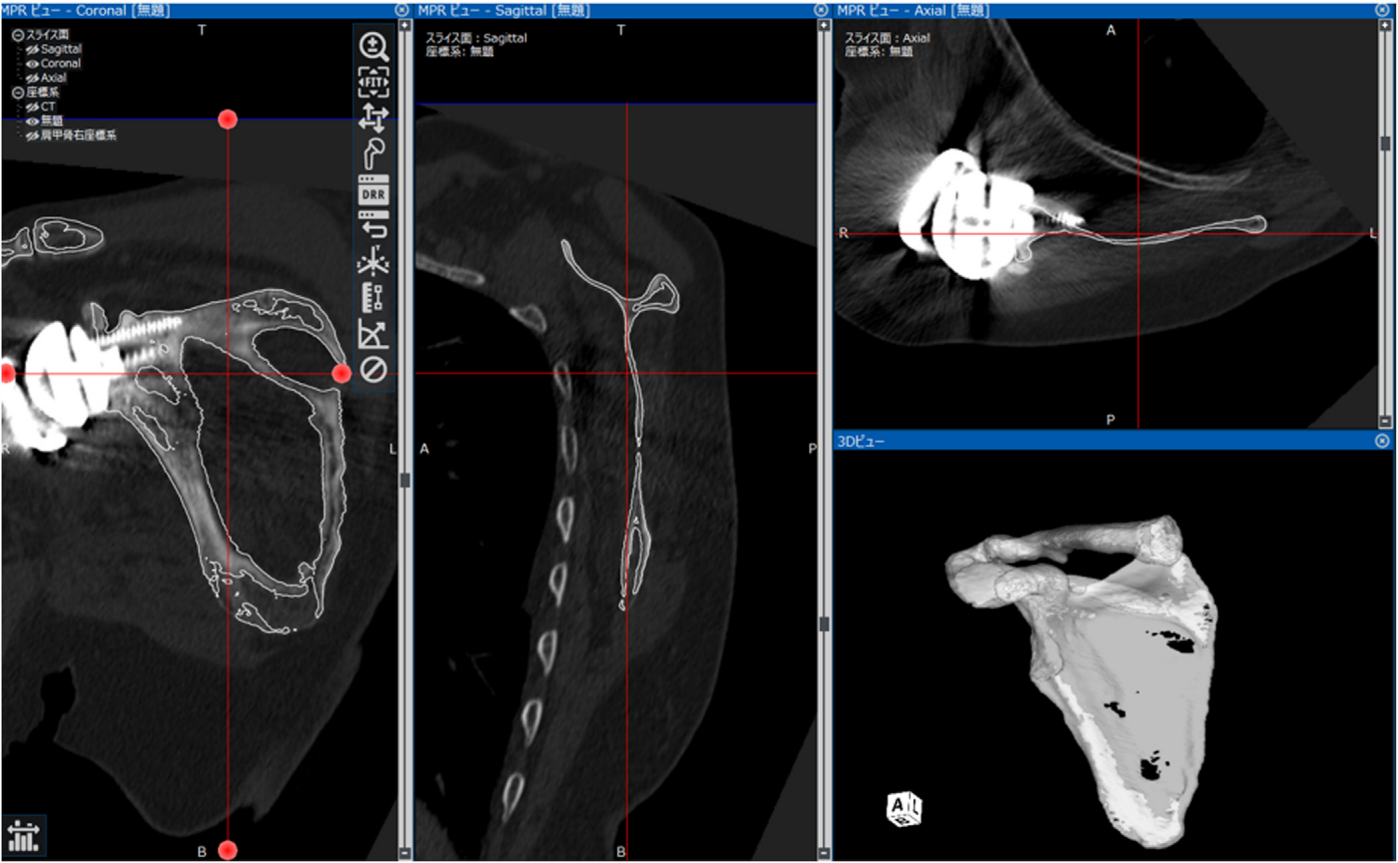
The method of superimposing postoperative CT and preoperative data. The coordinate system is constructed by superimposing the preoperative data on the postoperative CT images. *Red line*: lines to create multiplanar reconstruction; *White line*: preoperative data. *CT*, computed tomography.

**Figure 3:**
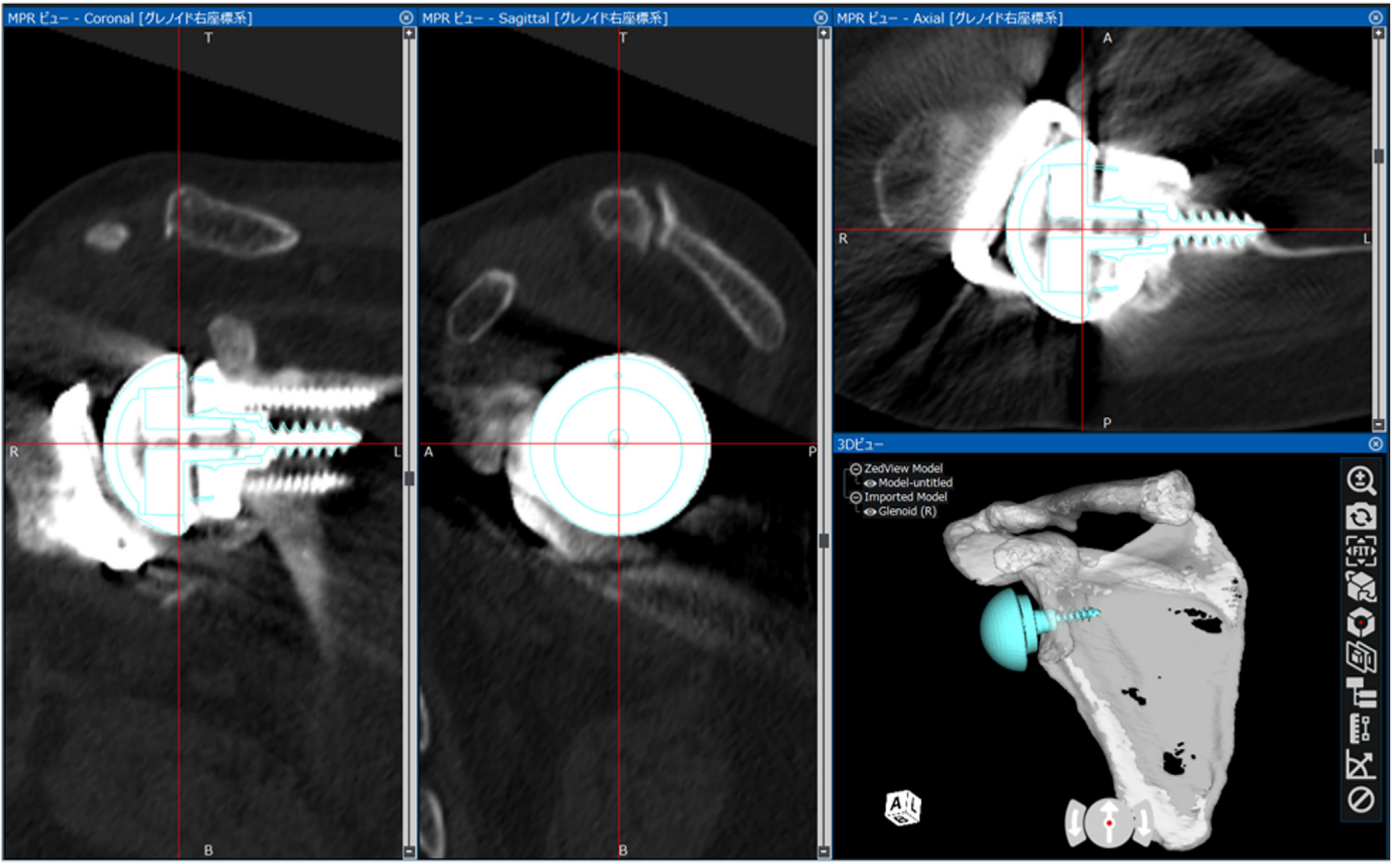
Implant matching method Zimmer Biomet's Comprehensive Reverse Shoulder System computer-aided design models were matched to the implants post-CT. *Red line*: line to create multiplanar reconstruction, *Blue line*: computer-aided design model of the Comprehensive Reverse Shoulder Arthroplasty System (Zimmer-Biomet, Warsaw, IN, USA). *CT*, computed tomography.

A 3D model was created from the postoperative CT images and superimposed into the preoperative data (Fig. 2). CAD models from Zimmer Biomet's Comprehensive Reverse Shoulder System were manually matched to the implant site in the postoperative CT data and evaluated (Fig. 3). A plane connecting the midpoint of the glenoid width, medial border of the scapula, and the inferior angle of the scapula was created. The X-axis was defined as the midpoint between the medial border of the scapula and glenoid width; the Z-axis was defined as the point perpendicular to the X-axis on the plane; and the Y-axis was defined as the point perpendicular to the X- and Z-axes.

In the present study, instead of CAD data for the VRS, a 25-mm base plate was used, and the glenosphere used a 36-mm B-type standard-type template (Fig. 3). GV and GI were measured automatically. GV was measured from the angle formed by the line connecting the medial border of the scapula and the anteroposterior midpoint of the baseplate and the line perpendicular to the baseplate. GI was measured from the angle formed by the line connecting the medial border of the scapula and the superior-inferior midpoints of the baseplate and the line perpendicular to the baseplate.

This measurement was performed by an independent observer.

### Statistical analysis

Data analysis included descriptive statistics, such as mean, standard deviation, and 95% confidence intervals. Intraclass correlation coefficients (ICCs) were calculated using standard statistical methods (ICC 1, 1; intra-observer reliability). The ICCs were classified as slight (≤0.20), fair (0.21 –0.40), moderate (0.41–0.60), substantial (0.61–0.80), or excellent (0.81–1.00). The ICCs for intraobserver reliability were calculated using postoperative GV and GI data.

## Results

The mean preoperative GV was 3.5 ± 36.8° (−44.1–42.2) and mean GI was 7.3 ± 23.3° (−32.4–36.9). The mean operative time was 131.4 ± 48.9 (80–206) min, and the mean intraoperative blood loss was 161.1 ± 94.2 (30–300) ml (Table I). Boss implants were used in two cases. No intraoperative complications, such as intraoperative fractures or blood transfusions, were observed.

### Placement of the baseplate

**Figure 4:**
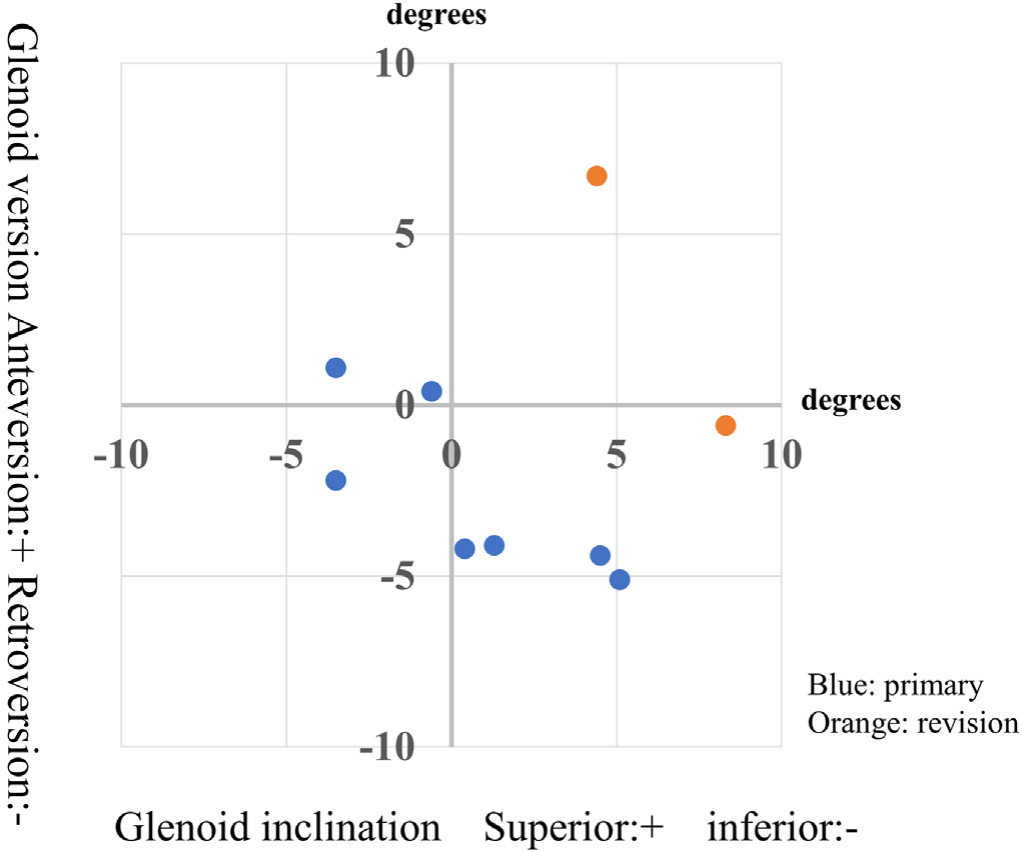
Error in postoperative position; *Blue*: primary cases; *Orange*: revision cases.

The interobserver reliabilities of GV and GI were 0.860 (0.470–0.965) and 0.887 (0.606–0.973), respectively, and they were excellent. The postoperative GV and GI values for each patient are shown in Table I. The range of error was 3.5 ± 2.5° (0.4–8.3) for GV and 3.2 ± 2.2° (0.4–6.7) for GI in absolute values. In primary cases, GV and GI errors from the target values were within 5° in six of seven cases (85.7%); in the revision cases, GV and GI were within 10° (Fig. 4).

## Discussion

In the present study, using VRS as CAD or CAM, the range of error of GV and GI was within 5° in 85.7% of primary cases and within 10° in revision cases. This study examined surgeries performed at different facilities and demonstrated that VRS placement was highly accurate.

The placement of the glenoid component is important, and malposition is known to lead to instability, scapular notching, and base of the acromion fractures and catastrophic failures in rTSA.^12^^,^^18^^,^^24^^,^^30^ In particular, superior tilt of the baseplate is associated with early component loosening and increased scapular notch.^20^ However, the scapula has few anatomic landmarks, making proper placement difficult.^19^ Particularly in cases of glenoid bone loss, scapular morphology is difficult to ascertain, leading to poor placement of implants. ^20^ The VRS, which is CAD or CAM, allows the use of guides that are CAM intraoperatively and is useful in avoiding these implant malpositions.

Bone grafts and augmented implants have been used to treat cases of glenoid bone defects.^3^^,^^21^^,^^28^ However, micromotion may occur when the contact area between the baseplate and bone is <50%.^6^ Bone grafts have unsatisfactory functional outcomes, radiographic loosening, graft subsidence, and graft resorption.^17^^,^^26^ Complications of bone grafting also include fractures, acute and chronic donor site pain, postoperative hematomas, nerve injuries, wound infections, and poor cosmesis.^1^^,^^5^^,^^21^^,^^25^ Moreover, although there are many reports of the usefulness of augmented implants in cases of glenoid bone defect, they may not provide adequate replacement in cases of severe bone defects owing to the limited amount that can be corrected per implant.^23^

In recent years, the usefulness of CAD or CAM implants in cases of severe glenoid bone loss has been widely reported.^7^^,^^9^^,^^11^^,^^14^^,^^27^ Gunther et al used this technique in seven patients with severe glenoid bone defects in TSA cases and reported that, at an average follow-up of 4.3 years, the American Shoulder and Elbow Surgeons score improved to 68 points and ROM (forward flexion, 33°; external rotation, 34°; internal rotation, 6 spinal levels), and the pain score improved from 6.9 to 0.1. In addition, there was no shift or subsidence of the glenoid implant as a radiographic change.^14^ Short-term results of the VRS used in this study were also reported by Bodendorfer et al. With a mean follow-up of 30 months, 12 shoulders were included in the study; seven were primary cases and five were revision cases. There were statistically significant improvements in median ROM measurements (forward elevation, 20°; external rotation, 40°; internal rotation, 2 spinal levels). In particular, they reported no evidence of implant loosening in primary and revision cases.^7^

In the present study, there was little error from the target placement position in primary and revision surgeries; in all cases, there was no superior tilt. These results support the low postoperative loosening rate of VRS and our conclusion that VRS is useful in both primary and revised cases of glenoid bone defects. However, in the revision case, the shape of the glenoid fossa after removal of the glenoid component was more challenging to predict preoperatively than in the primary case, and there were more errors in the placement of the guide, resulting in a poorer placement position than in the primary case. In addition, all three facilities performed adequate glenoid exposure and soft tissue removal for accurate placement with the VRS, which may have also affected the results.

The first limitation of this study was that it was a small case series. Second, the clinical outcomes were not evaluated. The VRS was introduced in Japan in June 2020, and the clinical score was evaluated during a 2-year follow-up period. However, a long-term follow-up is planned to investigate the relationship with radiographic outcomes, such as loosening. Third, there was no control group in this study because cases of severe glenoid bone defects had various glenoid morphologies and bone grafting methods, making it difficult to establish a control group. Fourth, surgery was performed by an experienced surgeon, and the evaluation was not based on the years of experience. Fifth, the CT imaging conditions varied among the facility, which may have influenced metal halation. However, in the present study, CT imaging was performed under conditions of reduced halation at all facilities, and good reproducibility was obtained in ICC evaluation.

To the best of our knowledge, this is the first study to evaluate the postoperative placement of a VRS using a CAD or CAM system in three dimensions. Using VRS, the range of error of GV and GI was within 5° in 85.7% of the primary cases and within 10° in the revision cases. Based on these findings, the placement position of the glenoid component in rTSA using VRS has a high reproducibility and is considered useful in cases of severe glenoid bone defects

## Conclusion

This is the first study to evaluate the postoperative placement of a VRS using a CAD or CAM in three dimensions. Using VRS, the range of error of GV and GI was within 5 °in 85.7% of the primary cases and within 10 °in the revision cases. Based on these findings, the reproducibility of the glenoid component placement position in the rTSA using VRS is high and is considered useful in cases of severe glenoid bone defects.

## Disclaimers:

Funding: No funding was disclosed by the authors.

Conflicts of interest: The authors, their immediate families, and any research foundations with which they are affiliated have not received any financial payments or other benefits from any commercial entity related to the subject of this article.
